# Sea Horse Optimization–Deep Neural Network: A Medication Adherence Monitoring System Based on Hand Gesture Recognition

**DOI:** 10.3390/s24165224

**Published:** 2024-08-12

**Authors:** Palanisamy Amirthalingam, Yasser Alatawi, Narmatha Chellamani, Manimurugan Shanmuganathan, Mostafa A. Sayed Ali, Saleh Fahad Alqifari, Vasudevan Mani, Muralikrishnan Dhanasekaran, Abdulelah Saeed Alqahtani, Majed Falah Alanazi, Ahmed Aljabri

**Affiliations:** 1 Department of Pharmacy Practice, Faculty of Pharmacy, University of Tabuk, Tabuk 71491, Saudi Arabia; yasser@ut.edu.sa (Y.A.); ma-ali@ut.edu.sa (M.A.S.A.); salqifari@ut.edu.sa (S.F.A.); 2 Faculty of Computers and Information Technology, University of Tabuk, Tabuk 71491, Saudi Arabia; narmatha@ut.edu.sa (N.C.); mmurugan@ut.edu.sa (M.S.);; 3 Department of Clinical Pharmacy, Faculty of Pharmacy, Assiut University, Assiut 71526, Egypt; 4 Department of Pharmacology and Toxicology, College of Pharmacy, Qassim University, Buraydah 51452, Saudi Arabia; v.samy@qu.edu.sa; 5 Department of Drug Discovery and Development, Harrison College of Pharmacy, Auburn University, Auburn, AL 36849, USA; dhanamu@auburn.edu; 6 Department of Pharmacy, NEOM Hospital, Neom 49626, Saudi Arabia; 7 Department of Pharmacy Practice, Faculty of Pharmacy, King Abdulaziz University, Jeddah 21589, Saudi Arabia; amaljabri@kau.edu.sa

**Keywords:** hand gestures, machine learning, medication adherence, sensor, smart wearable

## Abstract

Medication adherence is an essential aspect of healthcare for patients and is important for achieving medical objectives. However, the lack of standard techniques for measuring adherence is a global concern, making it challenging to accurately monitor and measure patient medication regimens. The use of sensor technology for medication adherence monitoring has received much attention lately since it makes it possible to continuously observe patients’ medication adherence behavior. Sensor devices or smart wearables utilize state-of-the-art machine learning (ML) methods to analyze intricate data patterns and provide predictions accurately. The key aim of this work is to develop a sensor-based hand gesture recognition model to predict medication activities. In this research, a smart sensor device-based hand gesture prediction model is developed to recognize medication intake activities. The device includes a tri-axial gyroscope, geometric, and accelerometer sensors to sense and gather data from hand gestures. A smartphone application gathers hand gesture data from the sensor device, which is then stored in the cloud database in a .csv format. These data are collected, processed, and classified to recognize the medication intake activity using the proposed novel neural network model called Sea Horse Optimization–Deep Neural Network (SHO-DNN). The SHO technique is implemented to update the biases and weights and the number of hidden layers in the DNN model. By updating these parameters, the DNN model is improved in classifying the samples of hand gestures to identify the medication activities. The research model demonstrates impressive performance, with an accuracy of 98.59%, sensitivity of 97.82%, precision of 98.69%, and an F1 score of 98.48%. Hence, the proposed model outperformed the most available models in all the aforementioned aspects. The results indicate that this model is a promising approach for medication adherence monitoring in healthcare applications, instilling confidence in its effectiveness.

## 1. Introduction

The global population of older adults is increasing at an unparalleled rate, and older individuals are prone to developing chronic ailments [1], including hypertension, diabetes mellitus [2], and cardiovascular disorders. Patients must continue to be treated for these disorders through drug therapy, which can be challenging [3]. The issue of patients not following their prescribed medication regimens is a widespread concern in healthcare, particularly among those with long-term health issues [4]. Non-adherence can arise due to many factors, including patients forgetting medicine intake, confusion in dosage guidelines, or deliberately deciding not to comply with the specified treatment plan. These factors can result in poor health, less effective therapies, and higher medical costs. Furthermore, non-adherence could be particularly difficult in home-care environments because patients lack the same kind of assistance and monitoring from medical providers as they would receive in hospitals [5].

Approximately 50% of people globally do not follow their prescribed drug regimens, making it a global concern [6]. Medication adherence refers to taking recommended medications in accordance with the recommendations of healthcare experts and patients. It is a complicated human behavior crucial for effectively managing chronic health problems [7]. Studies have revealed forgetfulness as the primary cause of non-adherence to several long-term medications. Smartphone applications and other technologically based alerts have demonstrated positive results in enhancing medication adherence [8,9].

Various technologies encompassed within digital health tools have the potential to enhance patients’ well-being and provide appropriate treatment, which has a significant impact on the prevention and management of non-communicable illnesses [10]. Additionally, these tools play a crucial role in improving patient adherence to therapy. Integrating electronic healthcare (eHealth) into medicine administration and prescription is crucial for improving medication safety, therapeutic effectiveness, and overall health outcomes [11]. Furthermore, these technologies can substantially impact providing information, imparting knowledge, tracking progress, and inspiring patients [12]. In the context of eHealth, several devices can be utilized, such as telecommunication technology (telehealth), mobile phones (mHealth), text messaging, and wearable devices such as smartwatches or sensor devices. mHealth refers to using mobile phones and wireless devices to enhance health outcomes. It also addresses issues related to therapy, education, and adherence [13].

Sensor devices or wearables are fixed to people’s bodies to collect customized data in real-time and consistently observe a patient’s health condition and biological signals conservatively [14]. Advanced wearable devices with sensors can be used to develop algorithms that enable customized diagnosis and treatment, often known as precision health. Nevertheless, it is imperative to create algorithms based on data that accurately reflect the expected users of wearable devices with sensors [15]. As this research focuses on hand gesture recognition, a gyroscope (GYRO) and accelerometer (ACC) are the main sensors used to measure angular rates and inertial accelerations, respectively. Furthermore, most human activities require measurement of acceleration, angular velocity, or other multi-dimensional information.

**Figure 1:**
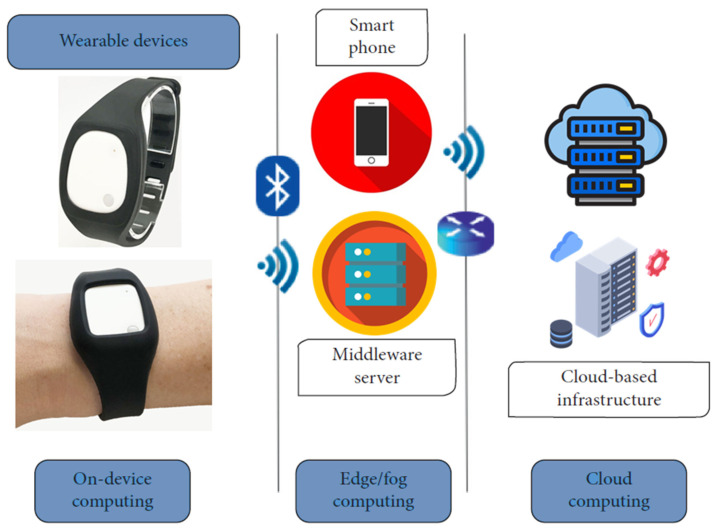
Application of Wearable Sensor Device.

Utilizing a solitary ACC or GYRO enables researchers to effectively observe and identify movement. Research has shown the potential to assess the intensity and categorize the movement of various activities, such as detecting falls, monitoring gait, and measuring physical activity [16]. Every sensor possesses distinct benefits and constraints; therefore, relying on only one sensor does not provide a thorough understanding of the information. Using a multi-sensor system enhances data dependability by offering redundancy. Wearable gadgets are physical manifestations of multi-sensor systems. These devices are equipped with various sensors that utilize different technologies. This enables them to perform various functions with different capacities [17]. Figure 1 depicts the model of wearable device application in data collection.

### 1.1. Problem Statement

Smart sensor devices, which are worn on the wrist, are equipped with a motion-sensing GYRO and ACC to detect the behavior of patients when taking medication. The wearable GYRO and ACC detect and log patient movements corresponding to pre-programmed actions for taking medicine. The physical movements of patients may serve as a proxy or indicator for the act of taking medicine [18,19]. Despite the availability of various medication adherence monitoring methods, more accurate and efficient approaches are needed to ensure that patients consistently adhere to prescribed medication regimens. Existing methods often lack real-time monitoring capabilities and may not effectively address individual patient needs, leading to suboptimal health outcomes and increased healthcare costs. Therefore, there is a pressing need to develop and evaluate innovative solutions that leverage advanced technologies, such as the proposed Sea Horse Optimization–Deep Neural Network (SHO-DNN) model, to improve medication adherence monitoring and enhance patient care in diverse healthcare settings based on hand gesture recognition.

### 1.2. Research Contribution

This study contributes to the advancement of medication adherence monitoring by proposing a novel SHO-DNN model. The model leverages sensor data collected from wearable devices to accurately predict medication adherence behaviors and offers real-time monitoring and personalized interventions to improve patient outcomes based on hand gestures. By integrating state-of-the-art optimization techniques and a machine learning (ML) algorithm, the proposed model provides a more robust and efficient solution for monitoring medication adherence in diverse healthcare settings. The research objectives were as follows:

- Use sensor-based devices for sensing and monitoring hand gestures to identify medication intake.
- Developing a novel SHO-DNN model for medication adherence monitoring based on hand gesture recognition.
- Update the hyperparameters of the DNN model using the SHO algorithm.
- Evaluate the performance of the SHO-DNN model using real-world sensor data.
- Measure the performance of the research model in terms of accuracy, sensitivity, F1-score, and precision.
- Compare and validate the performance of the SHO-DNN model with that of other current models.

### 1.3. Related Works

This section provides a detailed analysis of the related studies on sensor-based medication adherence systems. Inadequate adherence to medication leads to substantial economic consequences, including hospital readmissions, hospital visits, and additional healthcare expenses. For example, the study by Fozoonmayeh [20] used a cloud-based data pipeline and smartwatch application to construct a user-friendly method for tracking medicine consumption. The smartwatch application gathered activity sensor data using a GYRO and ACC. The data pipeline, which operated on the cloud, consisted of distributed storage of data, a management system for distributed databases, and distributed computing systems. These components were used to construct an ML model that accurately classified activity types based on sensor data. The study improved performance by effectively extracting and preprocessing sensor data and applying ML methods.

Digital systems may estimate adherence more effectively than manual techniques by minimizing human involvement and offering greater accuracy while reducing the user load. For instance, Kalantarian et al. [21] focused on the development of a system that employs smartwatches to identify adherence to prescription medicines. This was achieved by analyzing the motions detected by the built-in triaxial GYROs and ACCs. The effectiveness of the method was verified by analyzing the patterns of medicine intake and conducting experiments to classify movements.

Several smart pill bottle technologies can be used to identify when a container is opened and a pill is taken. However, only a few systems can identify if the medicine was consumed or not. To address this limitation, a smart necklace that detects drug ingestion based on skin movements in the lower neck during swallowing was proposed by Kalantarian et al. [22]. The system could identify more medication adherence use cases when combined with current medication adherence models that identify once the medicine is withdrawn. This system appropriately categorizes actions by using Bayesian networks.

In 2021, Wu et al. [23] created a prediction system that utilized environmental variables, lifestyle data, and patient symptoms to diagnose chronic obstructive pulmonary disease (COPD) within 7 days. This system relied on the use of sensors and wearable devices. The input characteristics were used to test the prediction performance of several ML models, including decision trees, random forests, k-nearest neighbors, adaptive boosting, linear discriminant analysis, and a DNN model. The DNN prediction model achieved an accuracy of 92.1%, a specificity of 90.4%, and a sensitivity of 94%.

A pipeline utilizing ML algorithms was developed by Moccia et al. [24] for the categorization of hand gestures. The pipeline uses wristband inertial sensors. This technique utilized a wristband sensor that incorporated a tri-axial GYRO and ACC. The evaluation process involved assessing deep learning (DL) and ML algorithms to classify multi-gesture and binary gesture categories based on inputs from a wristband sensor. The convolutional neural network (CNN) with long short-term memory yielded the best results, with an average F1-score of 90.5 for multi-gesture classification and 92.5 for binary classification.

Advanced sensor technologies have been developed to identify motions associated with taking pills, activating reminders, and providing adherence data. For example, Marquard et al. proposed that sensor-based solutions have the potential to facilitate customized reminders by utilizing objective real-time data on drug use [25]. An assessment was conducted on the medicine administration methods, physical movements, and feedback about the initial design. The adoption of these systems showed promise if the designs could fit a wide range of behaviors and preferences. These findings are consistent with the attitudes and behaviors of patients with several diseases where drug adherence is crucial.

A study by Kalantarian et al. [26] presented a system that could identify various movements related to medication adherence. This system uses a specialized Android application that operates on smartwatches. The time at which a pill container was opened and a medicine was taken was calculated utilizing a tri-axial GYRO and ACC. Notably, the system was compatible with any conventional twist-cap pill container, eliminating the need for sensors and wireless communication on each individual bottle, as was necessary with the Vitality Glowcap system. The classification accuracy of the wrist motion related to opening a pill container was significantly high and was achieved by utilizing three basic parameters from each axis of the ACC.

Lee and Youm [27] proposed a wearable camera image sensor to record patients taking drugs. CNN-based training was performed on the gathered data. This artificial intelligence approach to assessing medication behavior employed a faster region-based CNN object detection model (Model 1) and a model that uses integrated values of features for action identification (Model 2). Combining the two models resulted in a 92.7% accuracy rate for medication behavior recognition.

Ma et al. proposed a smartwatch application that used internal inertial sensors such as ACCs and GYROs to track medication intake activities [28] to increase adherence and deliver timely reminders. Data transmitted to the server were processed using Apache Spark and ML techniques to predict discrete behaviors such as medicine ingestion. These technologies preprocess the sensor data at a higher frequency and use the radio-frequency method, leading to increased recall and frequency.

Odhiambo et al. [29] conducted several trials on medication-taking behaviors. In the study, a smartwatch Android app was designed to gather hand-gesture data from a watch using an ACC. The acquired information was then transmitted to the main cloud database. A neural network model was created and subsequently trained using sensor data to accurately identify movements related and unrelated to medication adherence. Using the ML technique, the study achieved an average accuracy of 97% for the gesture data and 95% for normal data.

Effective monitoring plays a pivotal role in ensuring medication adherence. In a recent study by Aldeer et al. [30], a sensor-equipped pill bottle was used to identify medicine users. The bottle had a switch and inertial sensors on its top and body, offering discreet, cost-effective, and remote hardware access. Using the inertial data collected, researchers developed a patient discrimination model that employed classification techniques. The binary support vector machine (SVM) achieved a remarkable 94% accuracy in discriminating a single patient among 16 participants and 93% accuracy when using only one sensor. Furthermore, the model exhibited over 91% accuracy in precisely identifying individuals among three participants.

Capacitive sensing was used to create and test a static hand-motion detection system by Noble et al. [31]. The device utilized a 6 × 18 array of capacitive sensors to capture five distinct hand motions from five individuals, resulting in the production of gesture pictures. The dataset was employed to train various classification models, including Naïve Bayes, decision trees, multilayer perception (MLP) neural networks, and CNN classifiers. Each classifier underwent five training rounds, with four distinct individual gestures being used for training and one gesture for testing. The best classifier was MLP, with an accuracy rate of 96.87% and an F1 score of 92.16%.

An ACC-based off-body low-energy system with a low sampling rate was designed by Aldeer et al. [32]. Using an off-the-shelf motion sensor, a low-energy, accurate, and unobtrusive in-bottle patient-discrimination framework was built. The hardware included a three-axis ACC and a coin-cell-powered wireless transmission device. The software included pulse extraction, clustering, labeling, feature extraction, and classification. Subsequently, a discrimination model based on SVM effectively detected users, while binary SVM training and testing were conducted to identify the primary users. The model was tested with various user, training, and sensor counts. The algorithm accurately diagnosed patients using a single ACC, even with previously unknown participants.

Smart medication adherence monitoring systems can offer precise and detailed information on drug usage, allowing patients to participate actively in their treatment. Zijp et al. [33] evaluated the technical reliability and acceptability of a smart pill bottle prototype (SPBP) to facilitate further improvements. The evaluation assessed the rates of adherence, user approval, and technological robustness of an SPBP.

The SPBP was received positively, and the study yielded data used to enhance and conduct further optimization and follow-up investigations.

### 1.4. Research Gap

After reviewing the existing literature on medication adherence monitoring systems, it is evident that substantial progress has been made in utilizing various technologies such as smartwatches, smart pill bottles, inertial sensors, and wearable cameras coupled with ML and DL algorithms to track medication intake activities. However, as shown in Table 1, several research gaps still exist.

First, although many studies have focused on developing systems for detecting medication intake events based on sensor data, few have addressed the challenge of accurately distinguishing between actual medication ingestion and similar hand gestures or movements. Second, existing systems often rely on specific types of devices or sensors, which limit their applicability and scalability. Therefore, there is a need for more versatile and interoperable solutions that can be used in various devices and medication containers. Additionally, although some studies have achieved high accuracy in identifying medication adherence behaviors, there is a lack of real-world validation and deployment of these systems in clinical settings.

Thus, there is a gap in translating research findings into practical, scalable solutions that can be effectively integrated into healthcare workflows to improve medication adherence and patient outcomes. Furthermore, existing literature primarily focuses on technological aspects, with limited attention given to factors such as user acceptance, usability, and long-term engagement with these systems. Addressing these research gaps is crucial for developing more effective and user-friendly medication adherence monitoring solutions to positively impact patient health outcomes.

## 2. Materials and Methods

In this research, a medication adherence monitoring system is developed using a sensor device called MetaMotionR from Mbientlab, San Jose, CA, USA. This device monitors the hand gestures of a person consuming a medicine pill. The device included a tri-axial gyroscope, geometric, and accelerometer sensors to sense and gather data from hand gestures by taking medicines. A smartphone iOS application was used to gather hand gesture data from the device.

**Figure 2:**
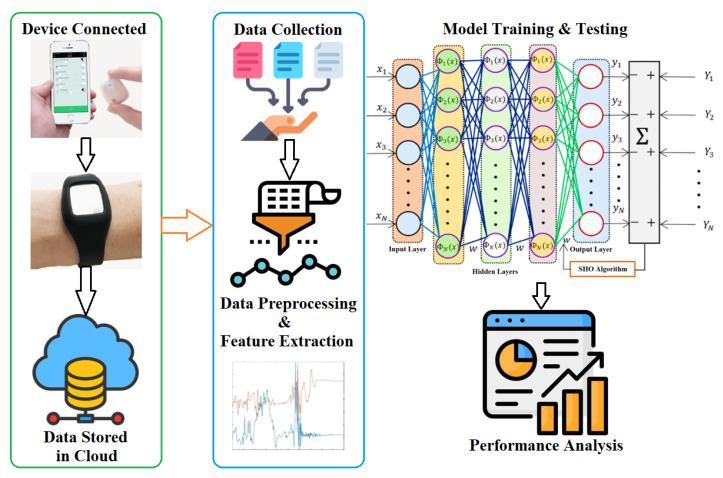
Pipeline of the Proposed Model.

The raw data from the sensor are saved in the cloud in a .csv format, which can be downloaded and accessed from anywhere using the app. In this research, a novel SHO-based DNN model was developed to classify the hand gestures, which is useful in properly identifying medication intake using the sensor data. The SHO technique was applied to update the two significant hyperparameters, biases and weights, and a number of hidden layers of the DNN model. Figure 2 represents the workflow of the proposed model.

### 2.1. Data Collection

The MetaMotionR device is perfectly suited for the purpose of recording and transmitting sensor data. Users can capture unprocessed sensor data using Bluetooth at a maximum frequency of 400 Hz. The unprocessed sensor data can be sent at a frequency of up to 100 Hz. The data can be downloaded and accessed by the user in the form of a .csv file on various devices, such as phones, tablets, computers, servers, or the cloud. Alternatively, the user can access the data as a JSON file utilizing the APIs offered by the manufacturer of the device.

**Figure 3:**
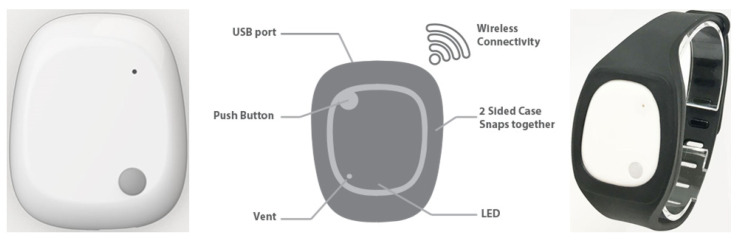
Visual Specification of MetaMotionR Device.

As shown in Figure 3, the device comprises a temperature sensor, a light sensor, NOR FLASH memory, and sensor integration with ten-axis motion sensing (including a three-axis accelerometer, a three-axis gyroscope, a three-axis magnetometer, and a barometer/altimeter/pressure sensor).

**Figure 4:**
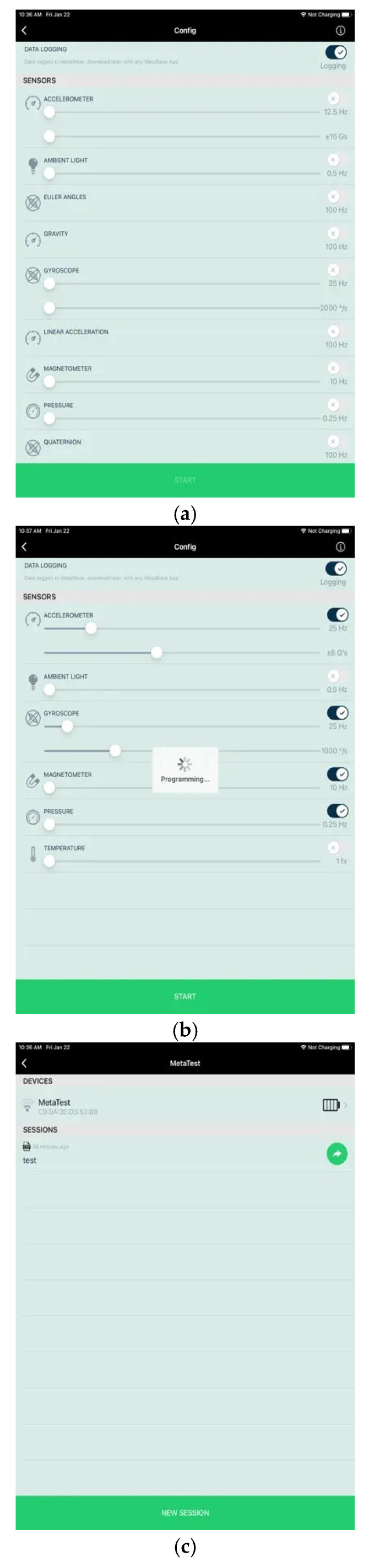
Data Logging, Sensors Selection, and Data Collection using MetaBase App.

As shown in Figure 4a–c, the application called “MetaBase” enables the user to set up the MetaSensors and access the data of the sensor. Figure 4a represents the selection of sensors; Figure 4b represents that the selected sensors are used for the application; Figure 4c represents the collected sensor data in a .csv format. This app is available for both Android and iOS smartphones. Using this device connected to this app, the hand gestures of opening a pill bottle, tipping a pill, tossing a pill to the mouth, holding a glass of water, and closing a pill bottle were monitored and recorded using the sensors in the device. Table 2 represents the hand gesture actions followed and monitored in this work.

### 2.2. Data Preprocessing

In preprocessing, data normalization is used to standardize numerical information prior to implementing clustering or classification methodologies that are primarily developed to manage numerical attributes. The normalization procedure is important to prevent some features from obscuring the impact of others, especially when the features have distinct ranges of variation. However, it is important to carefully choose the normalization technique and range (interval) during the preprocessing stage. This is because these choices have a substantial impact on the data and subsequently affect the results of the ML algorithm used after preprocessing. Z-score normalization is a common technique in the normalization process [34]. Z-score is a numerical approach used to address the problem of outliers. The average and standard deviations of the characteristics are utilized to convert the values of the features. To be more specific, the values for the features considered were converted into the newer normalized values utilizing Equation (1) [35].

(1) $$Z^{\prime }=\frac{Z-M}{SD}$$

In this equation, Z was the real value from the given data, $Z^{\prime }$ was the new normalized value, M represents the specified feature’s mean value, whereas SD represents the standard deviations of the features considered. By employing the Z-score approach, values that are lower than the mean are represented as negative numbers, values that are higher than the mean are represented as positive integers and values that were precisely equal to the mean were mapped to 0.

### 2.3. Sea Horse Optimization

The SHO is a unique metaheuristic that utilizes swarm intelligence. It was inspired by the natural movements, breeding, and predation behaviors of the sea horses. The suggested SHO technique has three essential components: movement, breeding, and predation. To achieve a balance between exploring and exploiting the SHO, specific search techniques were designed for the social behaviors of migration and predation, both at a local and global level. The breeding behavior is initiated upon the end of the initial two behaviors [36].

#### 2.3.1. Initialization

Like many established metaheuristic algorithms, SHO also begins with the initialization of the population. If all the sea horses in the search space of issues represent a potential solution, the whole sea horse population, denoted as Seahorses, could be represented as given in the following Equation (2):

(2) $$Seahorses=\left[\begin{array}{ccc} x_{1}^{1} & \cdots & x_{1}^{Dm}\\ \vdots & \ddots & \vdots \\ x_{pp}^{1} & \cdots & x_{pp}^{Dm} \end{array}\right]$$

Here, Dm represents the dimension of the variable, whereas pp refers to the population size. Every solution is produced randomly inside the specified problem’s upper bounds (UB) and lower bounds (LB). The *i*th individual $X_{i}$ in the search space [LB, UB] was expressed as in Equation (3):

(3) $$\begin{array}{c} X_{i}=\left[x_{i}^{1},\ldots ,x_{i}^{Dm}\right]\\ x_{i}^{j}=rand\times \left({UB}^{j}-{LB}^{j}\right)+{LB}^{j} \end{array}$$

In this equation, rand represents the random value that lies inside the scale of 0 to 1. The term $x_{i}^{j}$ represents the jth dimension in the ith person. The variable i represents a positive integer that can take values from 1 to the maximum value of pp. The variable *j* represents a positive integer that can take values from 1 to the value of Dm. ${UB}^{j}$ and ${LB}^{j}$ are the maximum and minimum values, respectively, for the *j*th variable in the optimized problem. Consider the minimal optimization issue, where the individual with minimal fitness was considered the elite individual, represented as $X_{elite}$. The variable $X_{elite}$ can be acquired using Equation (4).

(4) $$X_{elite}=argmin\left(f\left(X_{i}\right)\right)$$

Here, $f\left(\cdot \right)$ denotes the objective function value for the particular issue [37].

#### 2.3.2. The Behavior of Sea Horse Movement

The initial behavior of sea horses is characterized by movement patterns that closely adhere to a normal distribution with an SD of 1 and a mean of 0, as represented by the function $randn\left(0,1\right)$. To balance the trade-off between exploitation and exploration performances, it is assumed that $r_{1}=0$ serves as the threshold. Half of the resources are allocated to local mining, while the remaining half is dedicated to global search. Therefore, movement could be categorized into two scenarios.

Scenario 1: Sea horse spiraling with sea vortex. It mainly exploits SHO locally if the normal random value $r_{1}$ is the right section of the cut-off points. The spiral motion moves sea horses toward the elite $X_{elite}$. In particular, the Lévy flight simulates sea horse movement step size, which stimulates early moving to different places and avoids excessive local SHO utilization. The spiral-moving sea horse continually modifies its rotation angles to increase the neighborhoods of the current local solution. A new sea horse position could be calculated using Equation (5).

(5) $$X_{new}^{1}\left(t+1\right)=X_{i}\left(t\right)+Levy\left(\lambda \right)\left(\left(X_{elite}\left(t\right)-X_{i}\left(t\right)\right)\times x\times y\times z+X_{elite}\left(t\right)\right)$$

The equations $x=\rho \times cos\left(\theta \right)$, $y=\rho \times sin\left(\theta \right)$ and z=ρ×θ represent the three-dimensional components of the coordinates (x,y,z) in the spiral movements. The equations were helpful in updating the locations of search operators. The expression $\rho =u\times e^{\theta v}$ denotes the magnitude of the stems determined by the logarithmic spiral constant u and v (where u = 0.05 and v = 0.05). The variable θ was a random value that fell from 0 to 2π. The Lévy flight distribution function, $Levy\left(z\right)$, was computed utilizing Equation (6).

(6) $$Levy\left(z\right)=s\times \frac{w\times \sigma }{{\left|k\right|}^{\frac{1}{\lambda }}}$$

In Equation (5), λ was a random integer chosen from the interval [0, 2]. For this specific case, λ is set to the value of 1.5. The term ‘*s*’ is a constant with a 0.01 set value. The terms k and w were generated randomly on a scale of zero to one. The value of σ is determined by utilizing Equation (7).

(7) $$\sigma =\left(\frac{\Gamma \left(1+\lambda \right)\times \sin\left(\frac{\pi \lambda }{2}\right)}{\Gamma \left(\frac{1+\lambda }{2}\right)\times \lambda \times 2^{\left(\frac{\lambda -1}{2}\right)}}\right)$$

Scenario 2: Sea horse Brownian motion with sea waves. SHO exploration occurs when $r_{1}$ is left section of the cut-off points due to drifting. The search procedure was crucial for SHO local extreme avoidances. The Brownian movement simulates one more moving length to improve sea horse search space exploration. The equation for this situation is given in Equation (8).

(8) $$X_{new}^{1}\left(t+1\right)=X_{i}\left(t\right)+rand*l*\beta_{t}*\left(X_{i}\left(t\right)-\beta_{t}*X_{elite}\right)$$

Here, the constant coefficient l is set to a value of 0.05. The coefficient $\beta_{t}$ represents the random movement in Brownian motion. It follows a conventional normal distribution, meaning it is a random number with no specific pattern. Equation (9) could be utilized to obtain it.

(9) $$\beta_{t}=\frac{1}{\sqrt{2\pi }}exp\left(-\frac{x^{2}}{2}\right)$$

Indeed, these two scenarios could be expressed to derive the updated sea horse’s locations at t iteration in the following Equation (10).

(10) $$X_{new}^{1}\left(t+1\right)=\left\{\begin{array}{ll} X_{i}\left(t\right)+Levy\left(\lambda \right)\left(\left(X_{elite}\left(t\right)-X_{i}\left(t\right)\right)\times x\times y\times z+X_{elite}\left(t\right)\right) & r_{1}>0\\ X_{i}\left(t\right)+rand*l*\beta_{t}*\left(X_{i}\left(t\right)-\beta_{t}*X_{elite}\right) & r_{1}\leq 0 \end{array}\right.$$

**Figure 5:**
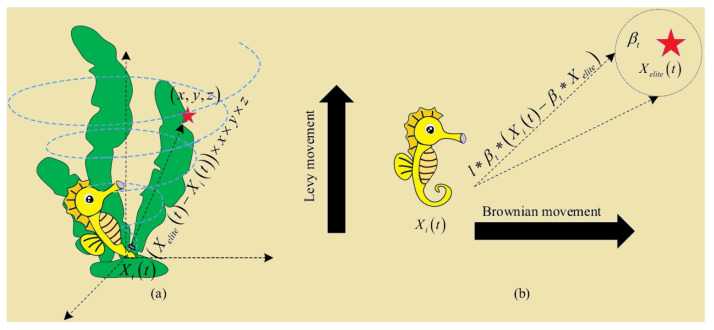
Patterns of DNN’s Spiral and Brownian Motions.

In this case, $r_{1}=randn()$ represents a random integer that follows a normal distribution. Figure 5 depicts the diagram for updating the positions of the sea horse. It shows two distinct movement modes: spiral (a) and Brownian motions (b). Both modes represent the sea horse’s random movement in response to the unpredictable marine environment.

#### 2.3.3. Sea Horse Predation Behavior

Sea horses that consume zooplankton and tiny crustaceans might succeed or fail. Since the sea horse’s chance of catching food is over 90%, SHO’s random number $r_{2}$ is set to 0.1 to separate these two findings. The elite shows the prey’s precise position. Therefore, SHO’s predation success accentuates its exploitation potential. If the value of $r_{2}$ is more than 0.1, it indicates that the sea horse’s predation was effective. Ineffective predation, the sea horses move up to the elite prey, move quicker than it, and catch it. If the predation fails, both change their reaction speeds, indicating the sea horse explores the search spaces. This predation behavior was expressed in Equation (11):

(11) $$X_{new}^{2}\left(t+1\right)=\left\{\begin{array}{c} \alpha *\left(X_{elite}-rand*X_{new}^{1}\left(t\right)\right)+\left(1-\alpha \right)*X_{elite}\;\;\;\;\;\;if\;r_{2}>0.1\\ \left(1-\alpha \right)*\left(X_{new}^{1}\left(t\right)-rand*X_{elite}\right)+\alpha *X_{new}^{1}\left(t\right)\;\;if\;r_{2}\leq 0.1 \end{array}\right.$$

Here, $X_{new}^{1}\left(t\right)$ represents the updated sea horse’s location following its movements during the t iteration. $r_{2}$ describes a random integer that lies on a scale of 0 to 1. The value of α decreases in a linear manner with each iteration to alter the step size of the sea horse while foraging for prey. This value is calculated using Equation (12).

(12) $$\alpha ={\left(1-\frac{t}{T}\right)}^{\frac{2t}{T}}$$

In this context, the variable T represents the maximum count of iterations. When a sea horse hunts effectively, it becomes exceptional. By increasing iterations and controlling parameter α, the system will progressively reach the global optimum individual. The global search was conducted because the prey is uncapturable. The 1−α parameter was implemented to the vectors among the elite and current individuals, and α affects the individual updated. This lets sea horses hunt broadly early on and prevent overexploiting later iterations.

#### 2.3.4. Sea Horse Breeding Behavior

The population is divided by fitness into men and women. As male sea horses procreate, the algorithm chooses fifty percent of the fittest individuals as mothers and the remaining as fathers. This divide would help fathers and mothers pass on positive characteristics to the next generation and prevent the over-localization of new solutions. Equation (13) is the succinct equation for the sea horse assignment task.

(13) $$\begin{array}{c} fathers=X_{sort}^{2}\left(1:pp/2\right)\\ mothers=X_{sort}^{2}\left(\frac{pp}{2}+1:pp\right) \end{array}$$

Here, $X_{sort}^{2}$ represents all $X_{new}^{2}$ arranged in ascending sequence depending on its fitness levels. The variables ‘mothers’ and ‘fathers’ represent the female and male population, accordingly. Male and female individuals were paired together in a random manner to create new offspring. To facilitate the execution of the technique, it was assumed that all the sea horse pairs engage in exclusive reproduction, resulting in the birth of a single offspring. The *i*th offspring’s expression is given as in Equation (14).

(14) $$X_{i}^{offspring}=r_{3}X_{i}^{father}+\left(1-r_{3}\right)X_{i}^{father}$$

Here, $r_{3}$ describes an integer randomly between the scale of 0 to 1, inclusive. The term i is a positive number that falls within the scale of $\left[1,pp/2\right]$. $X_{i}^{father}$ and $X_{i}^{father}$ denote individuals chosen randomly from the female and male population, respectively. 

**Figure 6:**
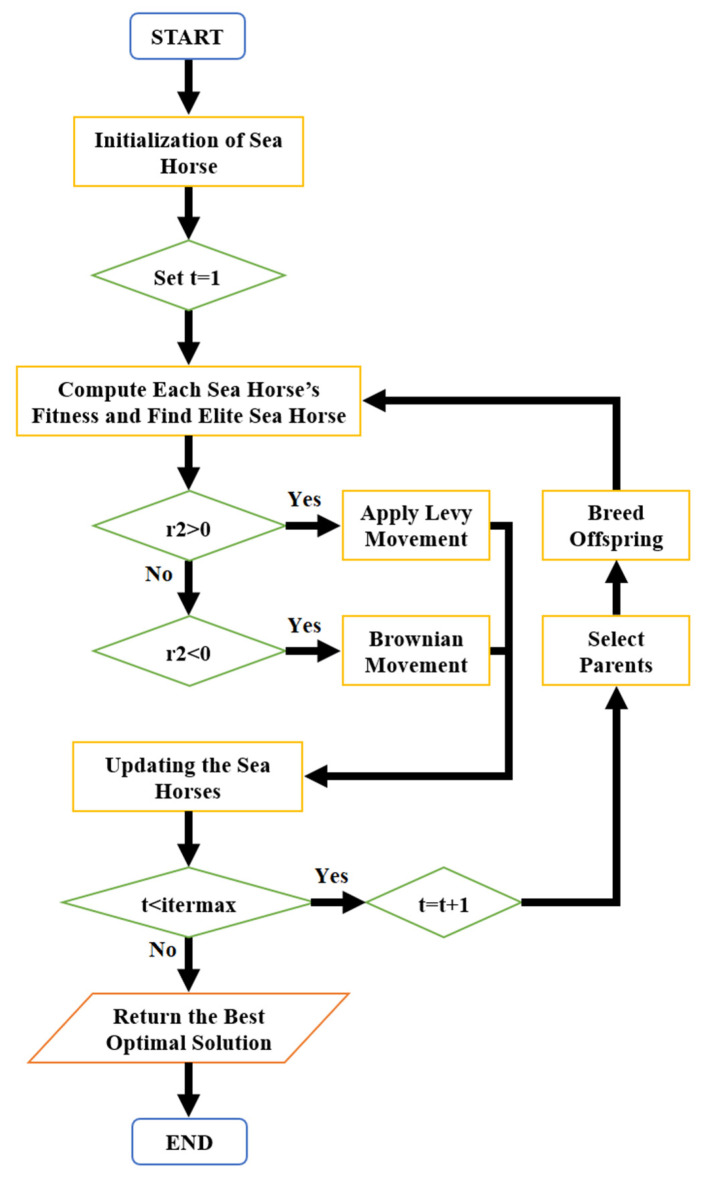
SHO Flowchart.

SHO implementation begins with population initialization using solutions randomly. Since updating the population of sea horses using Equations (10) and (11), Equation (12) breeds the offspring. Offspring and upgraded sea horses form a new population. However, this additional population is 1.5. To control population growth, each new individual is estimated. After sorting individuals by fitness ratings, the initial pp sea horses were repeatedly chosen as the new populations for the subsequent evolutionary phase [38]. Figure 6 depicts the flowchart of the SHO.

### 2.4. Deep Neural Network

**Figure 7:**
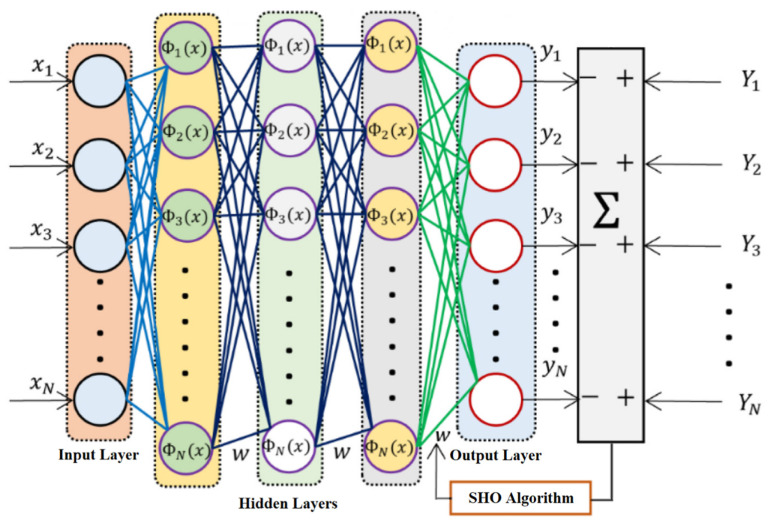
DNN Architecture.

The DNN is an ML model used to accurately represent intricate input-output interactions. The DNN architecture is a sort of supervised learning that includes several types of accelerators, convolution operations, matrix operations, and hidden layers. DNNs need a substantial number of hidden layers, rendering them very resilient. During the training process of a DNN, there are forward and backward passes. At all the iterations, the loss function was reduced using Gradient Descents (GD), Stochastic Gradient Descents (SGD), and adaptive moment estimations (Adam). DNN comprises fully linked layers along with activation, batch normalization (BN), and dropout techniques [39]. Figure 7 depicts the DNN architecture.

DNN is a hierarchical architecture composed of many layers of interconnected units. The units within each layer are not connected to each other. The model consists of an input layer and an output layer. In addition, many hidden layers were positioned within the input (*x*) and output layers (*y*). The total units in the input layers and output layers were determined based on the dimensions of the target data and input data. Nevertheless, there are no specific standards for configuring the count of units in all the hidden layers ($\Phi_{N}(x)$). The adjacent layers exhibit nonlinear interactions. In Equation (15), $a_{j}^{m}$ represents the neural unit *j*’s activation in layer m, the bias total and the previous activation’s linear combination was represented by $z_{j}^{m}$, and $b_{j}^{m}$ refers to the bias vectors associated with neural unit *j* in *m*. The matrix of weight $w_{ij}$ represents the connection strength between layers *i* and *j*. The activation function σ(z) was a mathematical function used to determine the output of a neural network. There are several types of activation functions available:

(15) $$a_{j}^{m}=\sigma \left(z_{j}^{m}\right)$$

(16) $$z_{j}^{m}=\sum_{i}w_{ij}a_{i}^{m-1}+b_{j}^{m}$$

Equation (16) represents a logistic function, which is primarily employed in DNNs. The function denoted by Equation (17) is commonly referred to as the hyperbolic tangent function. The output values range from −1 to 1. The logistic functions, although sometimes simpler to construct in neural networks, are distinct from the function being discussed. Equation (18) denotes the mathematical expression for the rectified linear unit (ReLU). The outcome of the system was consistently positive, as expressed in Equation (19). The work utilizes the logistic functions as the activation functions.

(17) $$\sigma \left(z\right)=1/\left(e^{-z}+1\right)$$

(18) $$\sigma \left(z\right)=\frac{e^{z}-e^{-z}}{e^{z}+e^{-z}}$$

(19) $$\sigma \left(z\right)=\max\left(0,z\right)$$

All the units in the subsequent layers were linked to every unit in the preceding layers. The activation unit in the input layers corresponds to the input data, whereas the output unit in the final layer corresponds to the objective values. The model’s parameters, w and b were initialized at random. Therefore, the values of y could be computed sequentially for each layer using the data of input x and the model’s parameters using the following Equations (20)–(22):

(20) $$a^{1}=x$$

(21) $$a_{j}^{m}=\sigma \left(\sum_{i}w_{ij}a_{i}^{m-1}+b_{j}^{m-1}\right),\;j>1$$

(22) $$y=a^{M}$$

Here, M represents the total levels of the networks. The first layer’s input, denoted as $a^{1}$, were the DNN’s input. The final layer’s output, denoted as $a^{M}$, were specified as the DNN’s output. To resolve the errors, the estimated outputs y can be compared with the target t using Equation (23):

(23) $$C\left(t,y\right)=\frac{1}{k}\sum_{k}\sum_{i}{\left(t_{i}-y_{i}\right)}^{2}$$

In the equation, C denotes the cost function, k refers to the total training samples, and i reflects the dimensions of y and t. The function of cost was employed to quantify the difference, and it often takes the structure of squared reconstruction errors. The DNN’s objective training was to decrease the function of cost, which entails reducing the inaccuracy of the network’s outputs to be as near to zero as possible. To reduce it, the GD technique was employed, and the gradient was evaluated using the following Equation (24):

(24) $$\nabla C=\left(\frac{\partial C}{\partial \theta_{1}},\frac{\partial C}{\partial \theta_{2}},\ldots ,\frac{\partial C}{\partial \theta_{N}}\right)$$

In the equation, the variable θ denotes the parameters, which encompass the biases and weights of the DNN model’s layers. The overall count of these parameters was represented by N. The partial derivative of the biases and weights of the last layer was initially computed based on the errors in the previous layers. Subsequently, the error was transmitted backward to previous levels, and each partial derivative was computed. After calculating the gradient, the parameters are modified according to the following Equations (25) and (26):

(25) $$w=w+\eta \frac{\partial C}{\partial w}$$

(26) $$b=b+\eta \frac{\partial C}{\partial b}$$

The variable η in the equations represents the learning rates. The computation of the gradients and the subsequent parameter update occur within an epoch. The epochs can be predetermined or dynamically modified based on the networks’ performance. The networks undergo training, during which the parameters are repeatedly adjusted until the network’s error reaches its lowest possible value [40,41].

### 2.5. Tuning DNN Hyperparameters Using SHO

When utilizing the DNN, it is imperative to carefully select the hyperparameter values. Table 3 provides a comprehensive list of the key hyperparameters and their corresponding values chosen for this research. This research specifically examined two crucial hyperparameters that exert a substantial influence on the performance of a DNN: the biases and weights associated with hidden layers and the number of hidden layers. It was well recognized that augmenting the number of hidden layers and neurons in the DNN model could enhance its capacity to represent information if overfitting is mitigated. Nevertheless, a substantial apprehension associated with increasing the number of neurons and hidden layers was the potential for overfitting. To mitigate the overfitting risk, a BN layer was used following all the subsequent hidden layers. BN layers have been demonstrated to substantially reduce training time by limiting internal covariance shifts. The SHO method is employed in this research to update the DNN’s biases and weights. (See Algorithm 1).
**Algorithm 1.** Pseudocode of SHO-DNNInitialize parameters of DNN weights (W) and biases (B) using random values Evaluate the fitness of each set of weights and biases Assign the best set of weights and biases as W_best and B_best Set the current iteration count t = 0 Set the maximum number of iterations T While (t < T)     If r1 = randn > 0      Then           Set v and u = 0.05           Rotation angle Rand (−2π, 2π)           Generate Levy coefficients            Update biases and weights of the DNN using SHO with W_best and B_best     Else           Set P = 0.05           Update weights and biases of the DNN using SHO     End if     Evaluate the fitness of each set of weights and biases using the training dataset     Select the best-performing sets of weights and biases as potential parents     Generate offspring by combining and mutating the selected parents     Evaluate the fitness of each offspring     Select the next iteration population from both parents and offspring     Update W_best and B_best if a better solution is found     Increment the iteration count t End while Return W_best, B_best

The DNN’s weights and biases are converted into a single population of solutions for the SHO algorithm. The SHO algorithm iteratively refines this population using seahorse-inspired movements, predator avoidance, and breeding strategies. After optimization, the best solution from SHO is decoded back into weights and biases, updating the DNN model. As shown in Figure 7, the SHO algorithm plays a significant role in optimizing the DNN model by fine-tuning the Weights and Biases of the network. The SHO process operates in the hidden layers, in which the algorithm iteratively adjusts the parameters to enhance the performance of the network in classifying hand gestures for monitoring medicine adherence. By updating the parameters of the network, the SHO algorithm enhances its performance, which results in accurate and reliable predictions of medicines’ intaking activities.

## 3. Results and Discussion

### 3.1. Experimental Setup

**Figure 8:**
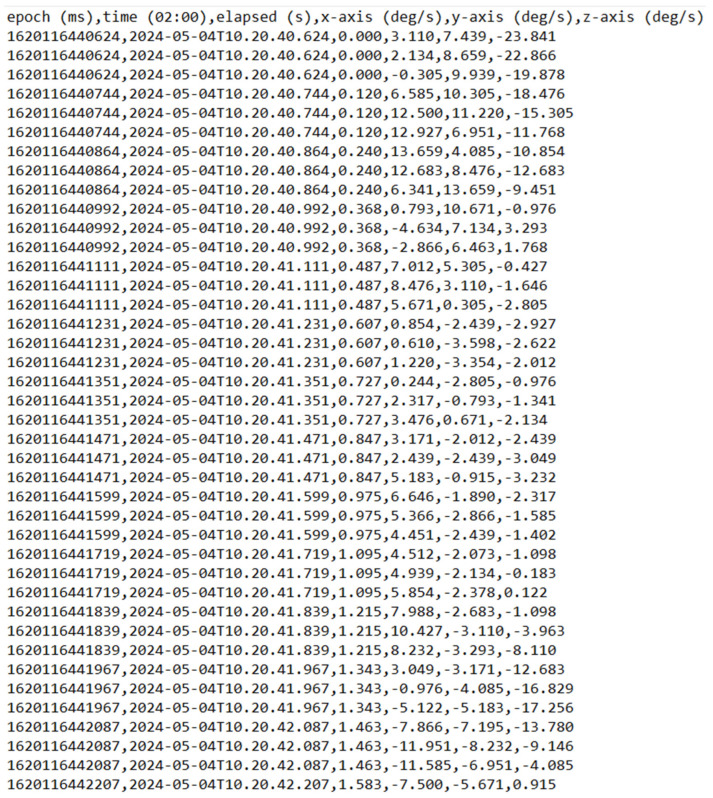
Visualization of Data Collected from the Sensor Device.

This section presents the results of the experiments carried out utilizing the SHO-DNN research model. The obtained sensor data are used to evaluate the experiment. The DNN model was developed using Keras 2.13.1 and Python 3.7.9, with TensorFlow 2.10 being used as the backend engine. The computer environment consisted of a Intel Core i7-620M central processor unit, 16 gigabytes of RAM, and a 64-bit version of the Windows 10 operating system. The data were split into an 80:20 ratio, where 80% of the data were used to train the model and the rest to evaluate. Table 4 represents the sensor data collected from the device. Figure 8 represents the raw data samples of hand gestures collected from the MetaMotionR sensor device.

### 3.2. Performance Metrics

The proposed SHO-DNN model’s results are evaluated based on accuracy, sensitivity, precision, and F1-score. Performance is assessed by considering the number of true positives, false positives, true negatives, and false negatives. The estimated performances are then compared with the existing models presented in the related works section to validate the research model’s performance.

Accuracy represents the proportion of properly classified samples among the total count of samples. It was calculated as the ratio of the number of proper predictions to the total count of predictions made. The formula for accuracy is presented in Equation (27):

(27) $$Accuracy=\frac{TP+TN}{TP+TN+FP+FN}$$

Sensitivity, sometimes referred to as recall or true positive rate, quantifies the ratio of accurately detected actual positive results by the model. It defines the ability of the model to properly identify positive instances from all actual positive instances. The formula for sensitivity is expressed in Equation (28):

(28) $$Sensitivity=\frac{TP}{TP+FN}$$

Precision represents the proportion of correctly identified positive cases among all instances predicted as positive by the model. It measures the model’s ability to avoid false positives. The formula for precision is presented in Equation (29):

(29) $$Precision=\frac{TP}{TP+FP}$$

F1-score is the harmonic mean of recall (sensitivity) and precision. It achieves a trade-off between recall and precision, considering false negative and false positive results. The F1-score achieves its optimal value at 1 and lowest value at 0. The F1-score is calculated using the following Equation (30):

(30) $$F1score=\frac{2\times Precision\times Recall}{Precision+Recall}$$

### 3.3. Performance Evaluation

This section presents the performance-evaluated results of the proposed SHO-DNN model based on the sensor data using the ten-fold cross-validation (CV) technique. Ten-fold CV was a commonly utilized technique in ML for assessing the performance of predictive models. It involves dividing the dataset into ten equal-sized subsets (folds), where each fold acts as a testing set once, while the rest of the nine folds were utilized for training the SHO-DNN model. This process was repeated ten times, with all the folds of the test set properly once. The average performance across all folds is then calculated to provide a more robust estimate of the model’s performance. Table 5 represents the results of the SHO-DNN model’s performance evaluated based on a ten-fold CV.

A ten-fold CV is essential for assessing a model’s generalization capability and detecting potential overfitting or underfitting issues. Repeatedly testing and training the model on diverse subsets of data helps assess how well the model performed on unseen data and provides more reliable estimates of performance metrics. The performance analysis table presents the results of the SHO-DNN model in each fold of the ten-fold cross-validation process.

**Figure 9:**
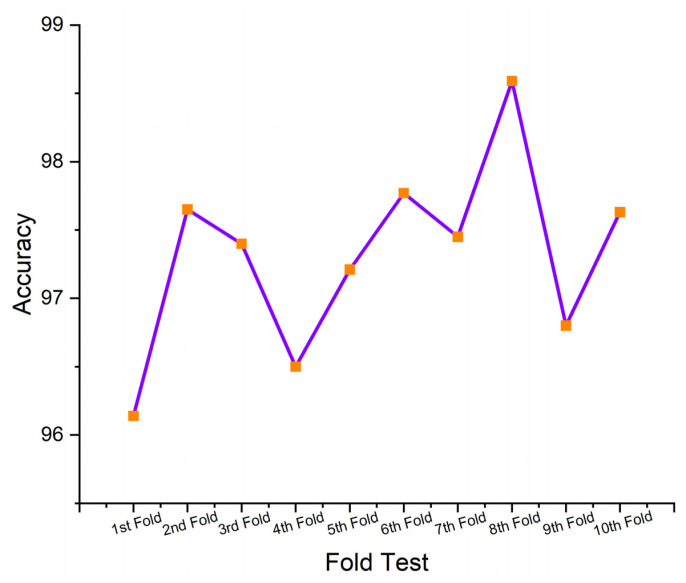
Graphical Plot of SHO-DNN Model’s Accuracy.

**Figure 10:**
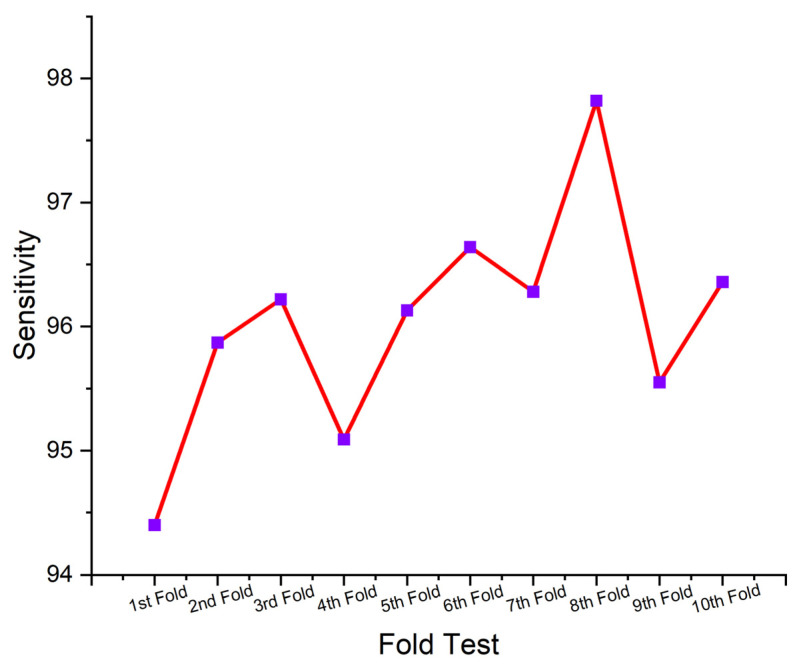
Graphical Plot of SHO-DNN Model’s Sensitivity.

**Figure 11:**
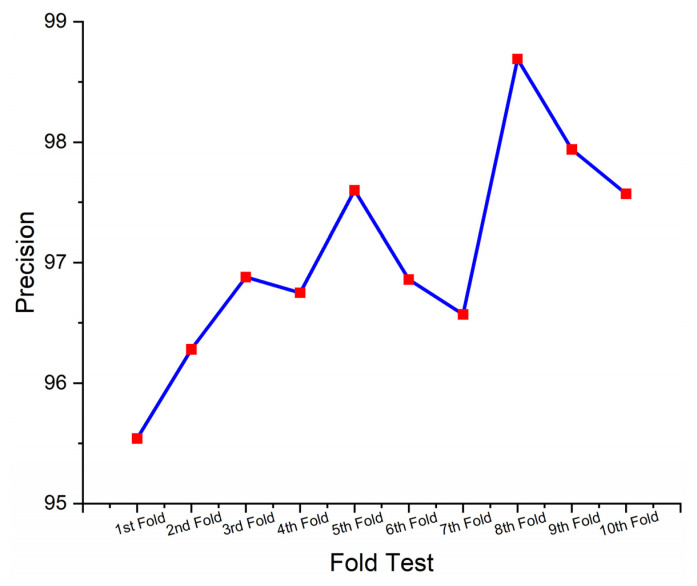
Graphical Plot of SHO-DNN Model’s Precision.

**Figure 12:**
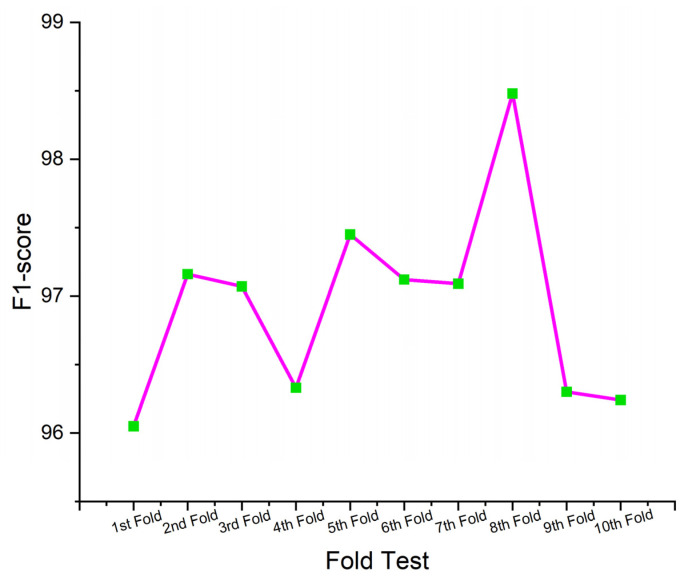
Graphical Plot of SHO-DNN Model’s F1-score.

Figure 9, Figure 10, Figure 11 and Figure 12 depict the graphical plots of the SHO-DNN model’s performance. The average accuracy of the model across all folds is approximately 97.18%. This indicates that the model correctly classifies about 97.18% of the instances in the dataset. The highest accuracy rate was attained in the 8th fold, with 98.59%. The average sensitivity of the model across all folds is approximately 96.38%. This suggests that the model effectively identifies about 96.38% of the true positive instances. The highest sensitivity rate was attained in the 8th fold with 97.82%.

The average precision of the model across all folds is approximately 97.04%. This indicates that among the instances predicted as positive by the model, about 97.04% are true positives. The highest precision rate was attained in the 8th fold, with 98.69%. The average F1-score of the model across all folds is approximately 97.01%. The F1-score provides a balance between recall and precision, and a high F1-score represents a better overall performance of the model. The highest F1 score was attained in the 8th fold, with 98.48%. The results in each test fold vary slightly, with accuracy ranging from 96.14% to 98.59%. Similarly, sensitivity, precision, and F1-score also exhibit slight variations across different folds. Overall, the model demonstrates consistent performance across all folds, with high values for accuracy, sensitivity, precision, and F1-score.

While the overall performance of the SHO-DNN remains higher in each test fold, minor fluctuations in performance parameters like accuracy, sensitivity, F1 score, and precision across different folds exist. These variations can be attributed to the differences in the composition of training and testing data in each fold and the inherent variability in the dataset. The consistency of the model’s performance across different folds indicates its robustness and ability to generalize well to unseen data, a crucial aspect of model evaluation and validation.

Table 6 presents the performance comparison of the SHO-DNN model with several other ML models discussed in the related works. The proposed SHO-DNN model achieves the highest accuracy of 98.59% among all models. It outperforms all other models, including Random Forest [23], AdaBoost [23], DNN [23], CNN [24], CNN-LSTM [24], Decision Tree [31], Bayesian Network [22], Gradient-Boost Tree [20], and MLP [31]. The SHO-DNN model demonstrates a substantial improvement in accuracy compared to other models. The model has an accuracy difference of 2.29% to 9.73%.

The proposed SHO-DNN model achieves a sensitivity of 97.82%, the highest among all models. It surpasses the sensitivity of other models, indicating its effectiveness in correctly identifying positive instances.

The SHO-DNN model shows a notable improvement in sensitivity compared to most other models. Its sensitivity rate difference is from 1.82 to 19.86%. The proposed SHO-DNN model achieves a precision of 98.69%, which is the highest among all models. It exhibits superior precision to other models, indicating its ability to avoid false positives.

The SHO-DNN model demonstrates a substantial improvement in precision compared to most other models. The precision rate difference is from 3.19 to 20.74%. The proposed SHO-DNN model achieves an F1 score of 98.48%, the highest among all models.

**Figure 13:**
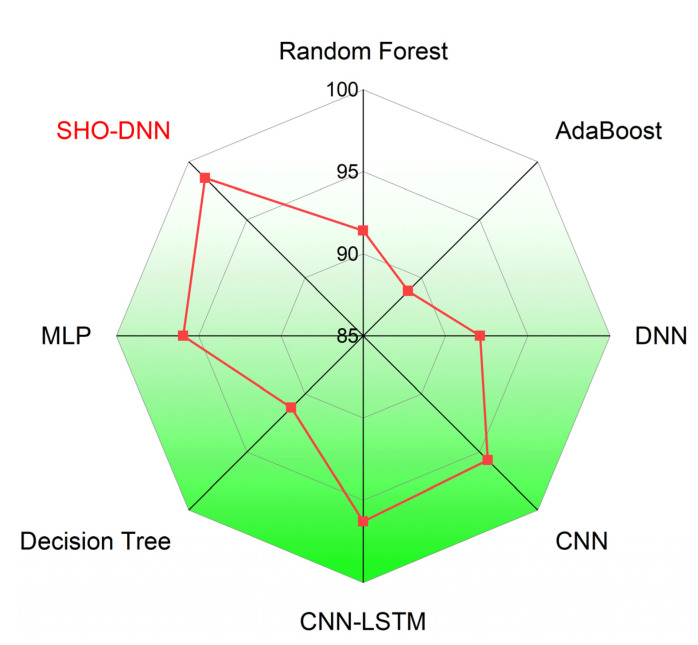
Graphical Plot of Accuracy Comparison.

**Figure 14:**
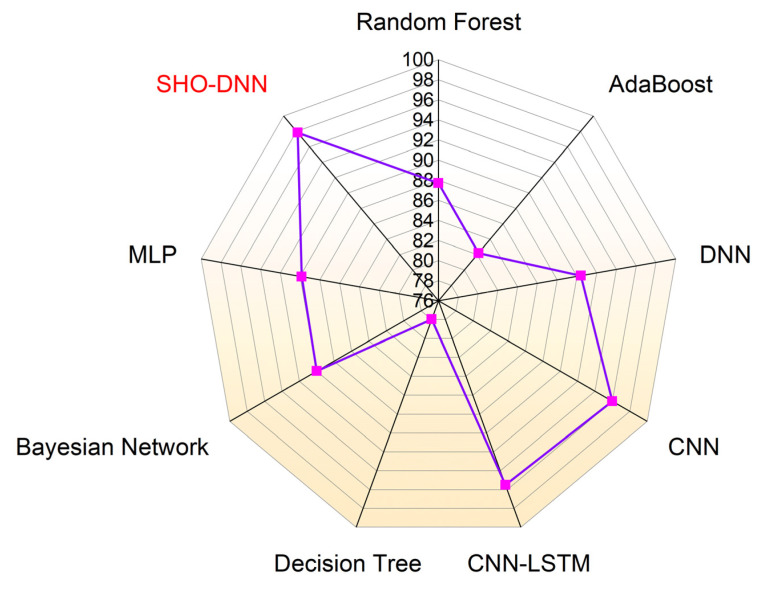
Graphical Plot of Sensitivity Comparison.

**Figure 15:**
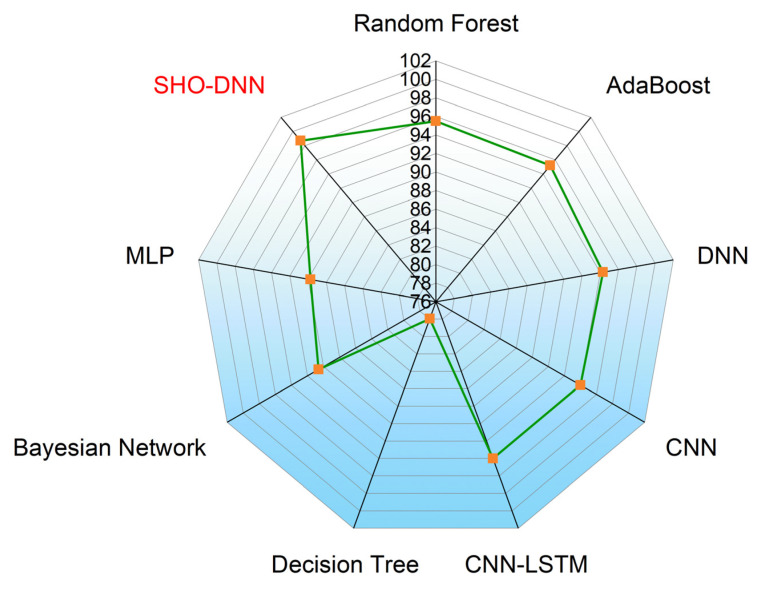
Graphical Plot of Precision Comparison.

**Figure 16:**
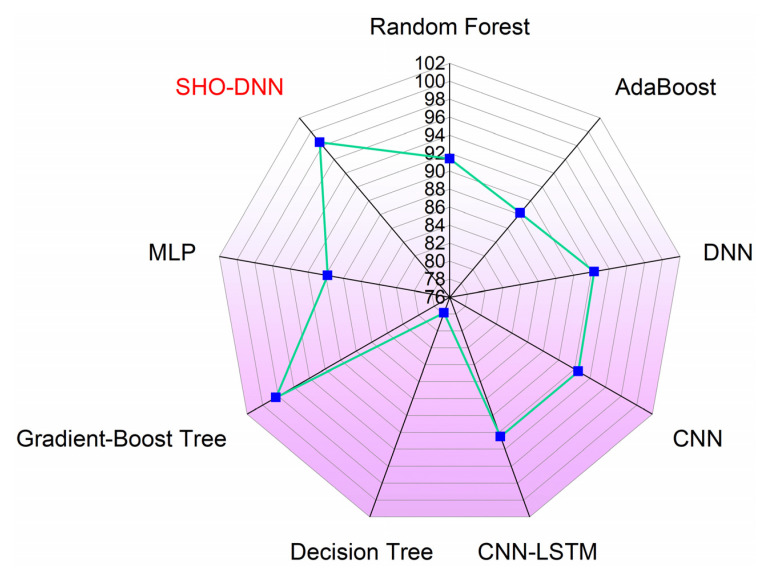
Graphical Plot of F1-score Comparison.

It outperformed every compared model in terms of the F1 score, indicating a balance between recall and precision. The SHO-DNN model shows a substantial improvement in the F1 score compared to other models. The model’s precision rate difference ranges from 0.18 to 20.63%. Figure 13, Figure 14, Figure 15 and Figure 16 represent the graphical plots of the compared performances individually. The SHO-DNN model outperforms all other models across all performance metrics. It exhibits substantial improvements in the results compared to other models, highlighting its effectiveness in predicting medication adherence.

The difference between the performance of the SHO-DNN model and other models varies, but generally, the SHO-DNN model shows a considerable improvement, ranging from a few percentage points to important margins.

Overall, the comparison results demonstrated that the SHO-DNN model achieved superior performance in predicting medication adherence compared to a variety of traditional and state-of-the-art ML models. Its robustness and effectiveness make it a promising approach for medication adherence monitoring in healthcare applications.

## 4. Conclusions

This research presents a medication adherence monitoring system based on a smart sensor device to sense and monitor hand gestures when taking medications. The sensor device monitored the hand gestures of a person taking a medicine pill. The device included a tri-axial gyroscope, geometric, and accelerometer sensors to sense and gather data from hand gestures such as opening a pill bottle, tipping a pill, tossing a pill to the mouth, holding a glass of water, and closing the pill bottle. A smartphone application was used to gather hand gesture data from the sensor device, which was stored in the cloud database in a .csv format. These data were collected, processed, and classified to recognize the medication intake activity using the proposed novel neural network model, SHO-DNN. Initially, the data were collected and preprocessed using the normalization technique to standardize the input data for the DNN model. After preprocessing, the data samples were processed and classified using the SHO-DNN model. The SHO algorithm was implemented to update the DNN’s weights and biases, as well as the hidden layers. By updating these parameters, the DNN model improved in classifying the samples of hand gestures to identify the medication activities. The performance of the SHO-DNN model was evaluated in terms of accuracy, sensitivity, F1 score, and precision based on a ten-fold CV. The research model attained 98.59% accuracy, 97.82% sensitivity, 98.69% precision, and a 98.48% F1 score. The results were compared with the current models for validation, and the model outperformed all other models. The SHO-DNN model demonstrates a substantial improvement in accuracy compared to other models. Overall, the comparison results demonstrate that the SHO-DNN model achieved superior performance in predicting medication adherence and turned out to be a promising approach for medication adherence monitoring in healthcare applications.

Future directions for this research include real-world deployment to assess feasibility and effectiveness in healthcare settings, exploration of additional sensor modalities for improved accuracy, longitudinal studies to evaluate long-term impact, optimization of user experience, and integration with healthcare systems to streamline medication management and enhance patient care coordination.

## Figures and Tables

**Table 1:** Comparative Analysis of Reviewed Existing Works.

Refs.	Approach Used	Application Scenario	Advantages	Disadvantages
[20]	Cloud-based data pipeline, Smartwatch application, ML model based on sensor data	Tracking medicine consumption, Activity classification	User-friendly method. Cloud-based architecture for scalability. Effective extraction and preprocessing of sensor data. Accurate activity classification.	Dependency on smartwatch and reliance on cloud infrastructure.
[21,22]	Smartwatch sensor analysis, Pattern recognition	Identifying adherence to prescription medicine	Utilizes built-in smartwatch sensors. Patterns of medicine intake analyzed. Reduced human involvement. Real-time monitoring.	Limited to specific smartwatch sensors. Not captured all medication intake scenarios.
[23]	Sensor data prediction, ML models	Diagnosing COPD	Utilizes environmental and lifestyle data. High accuracy in COPD prediction.	Limited to COPD prediction. Relies on sensor data availability.
[24,25,26]	ML and DL algorithms, Wristband inertial sensors	Gesture categorization	Utilizes wristband sensors. High classification accuracy. Effective for multi-gesture and binary classification.	Reliance on wristband sensors. Requires additional hardware.
[27]	Wearable camera image sensor, CNN-based training	Medication behavior assessment	Innovative use of wearable camera. High accuracy in medication behavior recognition.	Dependency on wearable camera. Privacy concerns.
[28]	Smartwatch internal sensors, Apache Spark, ML methods	Tracking medication intake activities	Utilizes smartwatch sensors. Scalable data processing with Apache Spark. High recall and frequency in behavior anticipation.	Limited to smartwatch users. Reliance on server infrastructure.
[29]	Smartwatch accelerometer data, Neural networks	Medication-taking behavior identification	Utilizes smartwatch sensors. High accuracy in gesture identification. Real-time data transmission.	Limited to smartwatch users. Requires continuous data transmission.
[30]	Sensor-equipped pill bottle, Inertial, and switch sensors	Identifying medicine users	Discreet and wireless hardware. High accuracy in patient discrimination.	Dependency on specific pill bottle hardware. Limited to medication adherence monitoring.
[31]	Capacitive sensing, ML classifiers (Decision Tree, Naïve Bayes, etc.)	Hand motion detection	Innovative use of capacitive sensors. High accuracy in gesture recognition.	Limited to static hand gestures. Relies on specific hardware.
[32]	Accelerometer-based system, SVM-based user discrimination model	Off-body patient identification	Low-energy and unobtrusive hardware. High accuracy in user discrimination.	Dependency on accelerometer. Not captured all user scenarios.
[33]	SPBP Adherence evaluation	Medication adherence monitoring	Real-world evaluation of SPBP. Positive user reception. Technical reliability assessment.	Limited to specific prototypes. Reliance on user acceptance.

ML: Machine learning, COPD: Chronic Obstructive Pulmonary Disease, DL: Deep learning, CNN: Convolutional neural network, SPBP: Smart pill bottle prototype.

**Table 2:** Step By Step Actions of Hand Gestures Monitored.

Step No.	Action	Description
1	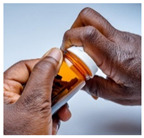	Opening the lid of the pill bottle by holding the bottle in one hand and rotating the bottle using a device-worn hand.
2	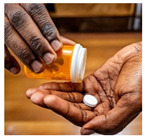	Tipping the bottle using a device-worn hand and dispensing a pill in another hand.
3	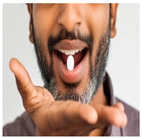	Tossing/placing a pill to mouth using a device worn by hand.
4	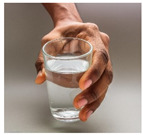	Holding a glass of water, using the device’s worn hand to consume water to swallow the pill.
5	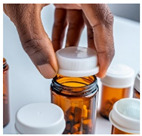	Closing the pill bottle using a device-worn hand.

**Table 3:** DNN Hyperparameters.

Parameters	Values
Optimization Algorithm	SHO
Number of Hidden Layers	3
Number of Biases and Weights	151
Number of Hidden Neurons	10
Dropouts	Nil
Output Layer Activation Function	Sigmoid
Hidden Layer Activation Function	Radial Basis

**Table 4:** Collected Data for Evaluation.

Feature	Value
Total Samples	4116
No. of Participants	15
No. of males	10
No. of females	5
Range of Age	26–32
Range of Weight	55–98 kg
Range of Height	151–182 cm

**Table 5:** Performance Analysis of SHO-DNN Model in Ten-Fold CV.

Fold Test	Accuracy	Sensitivity	Precision	F1-Score
1st Fold	96.14	94.40	95.54	96.05
2nd Fold	97.65	95.87	96.28	97.16
3rd Fold	97.40	96.22	96.88	97.07
4th Fold	96.50	95.09	96.75	96.33
5th Fold	97.21	96.13	97.60	97.45
6th Fold	97.77	96.64	96.86	97.12
7th Fold	97.45	96.28	96.57	97.09
8th Fold	98.59	97.82	98.69	98.48
9th Fold	96.80	95.55	97.94	96.30
10th Fold	97.63	96.36	97.57	96.24

**Table 6:** Comparison of SHO-DNN Model’s Performances.

Models	Accuracy %	Sensitivity %	Precision %	F1-Score %
Random Forest	91.40	87.70	95.50	91.40
AdaBoost	88.86	82.20	95.20	88.20
DNN	92.10	90.40	94.30	92.30
CNN	95.70	96.00	94.00	92.50
CNN-LSTM	96.30	95.50	94.00	92.50
Decision Tree	91.18	77.96	77.95	77.85
Bayesian Network	NA	90.00	90.62	NA
Gradient-Boost Tree	NA	NA	NA	98.30
MLP	95.94	89.84	89.75	89.77
SHO-DNN	98.59	97.82	98.69	98.48

## Data Availability

Data are contained within the article.
